# ATF3 regulates SPHK1 in cardiomyocyte injury via endoplasmic reticulum stress

**DOI:** 10.1002/iid3.998

**Published:** 2023-09-29

**Authors:** Huiling Chen, Suxin Luo, Huamei Chen, Cong Zhang

**Affiliations:** ^1^ Division of Cardiology The First Affiliated Hospital of Chongqing Medical University Chongqing P.R. China; ^2^ Division of Cardiology The First Affiliated Hospital of Kunming Medical University Kunming Yunnan P.R. China; ^3^ Department of Emergency The People's Hospital of ChuXiong YiZu Autonomous Prefecture Chuxiong Yunnan P.R. China

**Keywords:** ATF3, endoplasmic reticulum stress, myocardial infarction, oxidative stress, SPHK1

## Abstract

**Aim:**

Endoplasmic reticulum (ER) stress is common in different human pathologies, including cardiac diseases. Sphingosine kinase‐1 (SPHK1) represents an important player in cardiac growth and function. Nevertheless, its function in cardiomyocyte ER stress remains vague. This study sought to evaluate the mechanism through which SPHK1 might influence ER stress during myocardial infarction (MI).

**Methods:**

MI‐related GEO data sets were queried to screen differentially expressed genes. Murine HL‐1 cells exposed to oxygen‐glucose deprivation (OGD) and mice with MI were induced, followed by gene expression manipulation using short hairpin RNAs and overexpression vectors. The activating transcription factor 3 (ATF3) and SPHK1 expression was examined in cells and tissues. Cell counting kit‐8, TUNEL, DHE, HE, and Masson's staining were conducted in vitro and in vivo. The inflammatory factor concentrations in mouse serum were measured using ELISA. Finally, the transcriptional regulation of SPHK1 by ATF3 was validated.

**Results:**

ATF3 and SPHK1 were upregulated in vivo and in vitro. ATF3 downregulation reduced the SPHK1 transcription. ATF3 and SPHK1 downregulation increased the viability of OGD‐treated HL‐1 cells and decreased apoptosis, oxidative stress, and ER stress. ATF3 and SPHK1 downregulation narrowed the infarction area and attenuated myocardial fibrosis in mice, along with reduced inflammation in the serum and ER stress in the myocardium. In contrast, SPHK1 reduced the protective effect of ATF3 downregulation in vitro and in vivo.

**Conclusions:**

ATF3 downregulation reduced SPHK1 expression to attenuate cardiomyocyte injury in MI.

## Graphical abstract

Stimulation of activating transcription factor 3 (ATF3) exerts pro‐fibrotic and proinflammatory effect and expedite endoplasmic reticulum (ER) stress in cardiomyocytes via sphingosine kinase 1 (SPHK1). Thus, the inhibition of ATF3 and SPHK1 may have therapeutic potential for MI. Our understanding of ER stress during MI are still rudimentary, and more research is needed to support our conclusion.

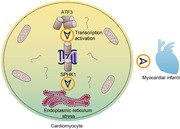

## 1 INTRODUCTION

Myocardial infarction (MI) is defined as the irreversible death of cardiomyocytes secondary to prolonged depletion of oxygen or blood supply.^1^, ^2^ The endoplasmic reticulum (ER) is an intracellular organelle, a part of the cellular reticular network that enables cells to respond to various conditions.^3^ ER stress initiates signal transduction events to reduce the accumulation of unfolded proteins, and if stress is severe and/or prolonged, ER can induce several apoptotic pathways that include the cleavage of caspase‐12.^4^ Initiation of the ER stress response has been linked to ischemic heart disease in mice.^5^ The ER redox environment governs the fate of entering proteins, and the expression of redox signaling regulators governs the generation of reactive oxygen species (ROS).^6^ Yet, the mechanism involved in ER stress and oxidative stress in MI is indefinite.

By querying two MI‐related GEO data sets: GSE46395 and GSE71906, we identified sphingosine kinase 1 (SPHK1) as the most significantly promoted gene in MI in the present study. SPHKs consist of a C‐terminal and an N‐terminal encompassing an ATP‐binding site and a sphingosine recognition site.^7^ Sphingosine‐1‐phosphate (S1P) is generated by phosphorylated SPHK1, and this intracellular signaling was involved in regulating ER stress, inflammation, and immediate early gene expression.^8^ It has been reported that following a 14‐day angiotensin II infusion SPHK1^−/−^ mice showed alleviated cardiac hypertrophy relative to wild‐type (WT) mice.^9^ Interestingly, SPHK1 upregulation is responsible for cerebral ischemia‐reperfusion injury by promoting ER stress and inflammation.^10^ Moreover, the SPHK1 protein expression was significantly upregulated following ischemic stress.^11^ Nevertheless, the specific impact of SPHK1 in modulating ER stress during MI needs further exploration. Mechanistically, SPHK1 expression has been revealed to be transcriptionally activated by FoxO3, a transcription factor, during cerebral ischemia/reperfusion injury.^12^ Therefore, we postulated that the transcription of SPHK1 was regulated by a transcription factor as well in MI. Activating transcription factor 3 (ATF3) was identified as a possible candidate for mediating the transcription of SPHK1 in MI in the work. ATF3 belongs to the AP‐1 family, which plays a part in cardiac remodeling.^13^ ATF3 expresses lowly in quiescent cells and highly under stress conditions, involving injury, ischemia, as well as ischemia/reperfusion, which makes it an adaptive‐response gene.^14^ Additionally, the knockout of ATF3 significantly aggravated ischemia/reperfusion injury, which could be rescued by ATF3 overexpression, which also changed the transcription levels of multiple ferroptosis‐related genes.^15^ In this article, we assessed whether ATF3 modulated oxidative stress and ER stress in MI by inducing SPHK1.

## 2 MATERIALS AND METHODS

### 2.1 Cell culture and treatment

Mouse cardiomyocytes HL‐1 were acquired from Tongpai and grew in minimal essential medium (MEM). 293 T cells were procured from the National Collection of Authenticated Cell Cultures and grew in Dulbecco's modified Eagle's complete medium. The complete media were supplemented with 10% fetal bovine serum and 1% penicillin/streptomycin in the basal medium. The culture environment was at 37°C with 5% CO_2_.

Plasmids sh‐SPHK1 (#1, #2, and #3), sh‐ATF3 (#1, #2 and #3), oe‐SPHK1, sh‐NC, and oe‐NC were acquired from VectorBuilder for cell transfection. HL‐1 cells were resuspended in MEM complete medium and plated into 6‐well plates. The cells were transfected with Hieff Trans^TM^ Universal Transfection Reagent (40808ES02, Yeasen) when they reached a 70% confluence. After 48 h, the cells were collected for the following experimental analysis.

To simulate the state of HL‐1 cells in MI, the cells were treated with oxygen‐glucose deprivation (OGD). Briefly, the cells were plated into cell culture plates after resuspension with a complete medium and placed in a 5% CO_2_ incubator at 37°C overnight. After the removal of the medium on the next day, the cells were supplemented with glucose‐free medium and incubated in a hypoxic chamber (5% CO_2_ and 95% N_2_) for 3 h.^16^ The cells cultured in a complete medium in a normoxic (5% CO_2_ and 20% O_2_) culture environment were set as a control group.

### 2.2 CCK‐8

After being plated into 96‐well plates, the viability of cells was assessed with cell counting kit‐8 (CCK‐8) (C0037, Beyotime) after HL‐1 cells were exposed to OGD. After the addition of 10 μL CCK‐8 for 1‐h incubation, a microplate reader was utilized to record the OD value at 450 nm.

### 2.3 TUNEL assay

TUNEL (T2190, Solarbio) assay was conducted for apoptosis evaluation of HL‐1 cells. The cells were fixed with 4% paraformaldehyde and fixed with Triton X‐100. TUNEL assay solution was configured for a 1‐h incubation at 37°C in the dark. The nuclei were treated with Hoechst. Finally, the apoptosis of the cells was viewed under a fluorescence microscope.

### 2.4 Dihydroethidium (DHE) staining

DHE (S0063, Beyotime) was applied for ROS measurement. DHE was diluted with dimethylsulfoxide, and HL‐1 cells were treated for 30 min at 37°C with 5 μM DHE in darkness. Finally, after DAPI for nuclear staining, the cells were viewed under a fluorescence microscope.

### 2.5 Determination of gene expression by qPCR

TRIzol (15596018, Invitrogen Inc.) was applied for the isolation of total RNA from cells and infarct margin tissues below the ligature line. The RevertAid First Strand cDNA Synthesis Kit (K1622, Thermo Fisher Scientific Inc.) was utilized after confirming the concentration and purity of the RNA. The cDNAs were subsequently quantified fluorescently using TB Green® Premix Ex Taq™ II (RR820Q, Takara). The mRNA primers are SPHK1, 5′‐GCTTCTGTGAACCACTATGCTGG‐3′ (F) and 5′‐ACTGAGCACAGAATAGAGCCGC‐3′ (R); ATF3, 5′‐GAAGATGAGAGGAAAAGGAGGCG‐3′ (F) and 5′‐GCTCAGCATTCACACTCTCCAG‐3′ (R); GRP78, 5′‐TGTCTTCTCAGCATCAAGCAAGG‐3′ (F) and 5′‐CCAACACTTCCTGGACAGGCTT‐3′ (R); Caspase‐12, 5′‐CAGATGAGGAACGTGTGTTGAGC‐3′ (F) and 5′‐GGAACCAGTCTTGCCTACCTTC‐3′ (R), β‐actin 5′‐CATTGCTGACAGGATGCAGAAGG‐3′ (F) and 5′‐TGCTGGAAGGTGGACAGTGAGG‐3′ (R). The relative mRNA levels were determined using the $2^{‐\Delta \Delta C_{t}}$ method and corrected for the β‐actin mRNA level.

### 2.6 Western blot

Radio immunoprecipitation assay Lysis Solution (R0010, Solarbio) was used for the isolation of proteins from cells and infarct margin tissues below the ligature line. Protein concentrations were subsequently measured with bicinchoninic acid assay protein assay kit (PC0020, Solarbio). Protein samples were separated by sodium dodecyl‐sulfate polyacrylamide gel electrophoresis and transferred to polyvinylidene difluoride membranes. Nonspecific binding sites were sealed with 3% skimmed milk for 45 min. The primary antibodies to SPHK1 (1:500, 10670‐1‐AP, ProteinTech Group), ATF3 (1:1000, ab254268, Abcam), CHOP (1:1000, #2895, Cell Signaling Technologies), ATF6 (1:1000, ab227830, Abcam), p‐IRE1α (1:500, PA1‐16927, Thermo Fisher Scientific), IRE1α (1:500, A17940, ABclonal), p‐eIF2α (1:1000, ab32157, Abcam), eIF2α (1:500, AHO0802, Thermo Fisher Scientific), and β‐actin (1:1000, 4970, Cell Signaling Technologies) was applied overnight (4°C), followed by re‐probing with HRP‐conjugated secondary antibodies (1:4000, ab6721, Abcam or 1:5000, #31430, Thermo Fisher) for 40 min at room temperature. Finally, the protein bands were measured using ECL (32209, Thermo Fisher).

### 2.7 ChIP

The 293 T cells were transfected with sh‐ATF3#1, #2, and #3. The mRNA expression of ATF3 in cells was detected by reverse transcription‐quantitative polymerase chain reaction (RT‐qPCR), and sh‐ATF3 #2 was selected for subsequent experimental analysis. The SimpleChIP® Enzymatic ChIP Kit (9003 S, Cell Signaling Technologies) was used for the determination of ChIP assays. The cells were treated with 1% formaldehyde at room temperature for 10 min and co‐treated with glycine for 5 min. The DNA was digested to a length of 150−900 bp as described in the kit's instructions. The chromatin complexes were subsequently immunoprecipitated with ATF3 (1:50, ab254268, Abcam) or its negative control rabbit IgG (1:100, ab171870, Abcam). DNA extraction was performed after de‐crosslinking with NaCl and Proteinase K. Finally, the enrichment of the SPHK1 promoter in the precipitated DNA was detected by qPCR.

### 2.8 Reporter assay

In hTFtarget (http://bioinfo.life.hust.edu.cn/hTFtarget/#!/prediction), the binding site of ATF3 and the SPHK1 promoter was predicted. The WT and mutant (MUT) binding sequences of the SPHK1 promoter to ATF3 were sub‐cloned into the pGL3‐basic plasmid to construct a luciferase reporter gene vector. After 24 h, the luciferase activity of the cells was assessed using the Dual Luciferase Assay Kit (11402ES60, Yeasen).

### 2.9 Mice

The Guide for the Care and Use of Laboratory Animals of the NIH was followed throughout the experiment regarding animals. The Committee of the Ethics of Animal Experiments of the First Affiliated Hospital of Chongqing Medical University authorized our protocol. Forty male C57BL/6J mice (8−9 weeks) were procured from Vital River (Beijing, China). For the MI modeling, the mice were treated with 2% isoflurane for anesthesia. The hearts were exposed through a left thoracotomy, and the LAD was ligated using a 6−0 silk suture to induce MI.^17^ Mice in the sham‐operated group were subjected to the same procedure but were not ligated.

For gene expression manipulation, the mice were injected with AAV after MI modeling. AAV‐sh‐SPHK1, AAV‐sh‐ATF3, AAV‐oe‐SPHK1, AAV‐sh‐NC, and AAV‐oe‐NC were procured from VectorBuilder. The mice were subjected to anesthesia 1 week after LAD ligation, and AAV were administered into the ischemic heart.^18^ The mice were euthanized by sodium pentobarbital at 150 mg/kg (*i.p*.) 4 weeks after modeling. The blood and heart tissues (the whole heart for histological staining and infarct margin tissues for expression measurement) of mice were harvested for subsequent experimental analysis.

### 2.10 Echocardiography

Mouse cardiac function was assessed by M‐mode echocardiography using the Vevo 2100 ultrasound imaging system (VisualSonics, Inc.). After the fourth week of modeling, the hair was removed from the chest, and the mice were anesthetized and placed on a heating pad. The heart was imaged in long‐axis and short‐axis views to measure left ventricular (LV) systolic and diastolic internal diameters, and the LV ejection fraction (LVEF) and fractional shortening (LVFS) were further measured. All measurements were performed by researchers who were unaware of the grouping.

### 2.11 Histological staining

Mouse whole heart tissues were fixed overnight in 4% paraformaldehyde, paraffin‐embedded, and sectioned after dehydration. The tissue sections were subsequently stained using HE (E607318; Sangon). The HE staining images were scored according to the degree of cardiomyocyte hypertrophy (0−3) and the degree of inflammatory infiltration (0−3), and the total score was the sum of the scores of the two aspects (0−6). For Masson staining, the Masson trichrome staining kit (G1346, Solarbio) was used according to the protocol. The infarct area was measured as the total infarct circumference divided by the total LV circumference.

### 2.12 Mouse cytokine array

The ELISA kits for TNF‐α (PT512, Beyotime), IL‐1β (PI301, Beyotime), and S1P (K‐1900, Echelon Biosciences) were used to detect their respective levels. Briefly, mouse serum and standards were supplemented to the assay plate as described in the kits. Finally, the standard curves were plotted according to the concentrations of the standards, and the contents of TNF‐α, IL‐1β, and S1P in the serum were calculated.

### 2.13 Statistics

Our data are expressed as means ± SD and analyzed using GraphPad Prism 8.0.2 software (GraphPad). The comparison of any two means was analyzed using the unpaired *t*‐test, and the comparison among several means was analyzed using one‐way or two‐way analysis of variance. followed by Tukey's analysis. *p* < 0.05 denotes significant changes.

## 3 RESULTS

### 3.1 SPHK1 is overexpressed in MI

**Figure 1:**
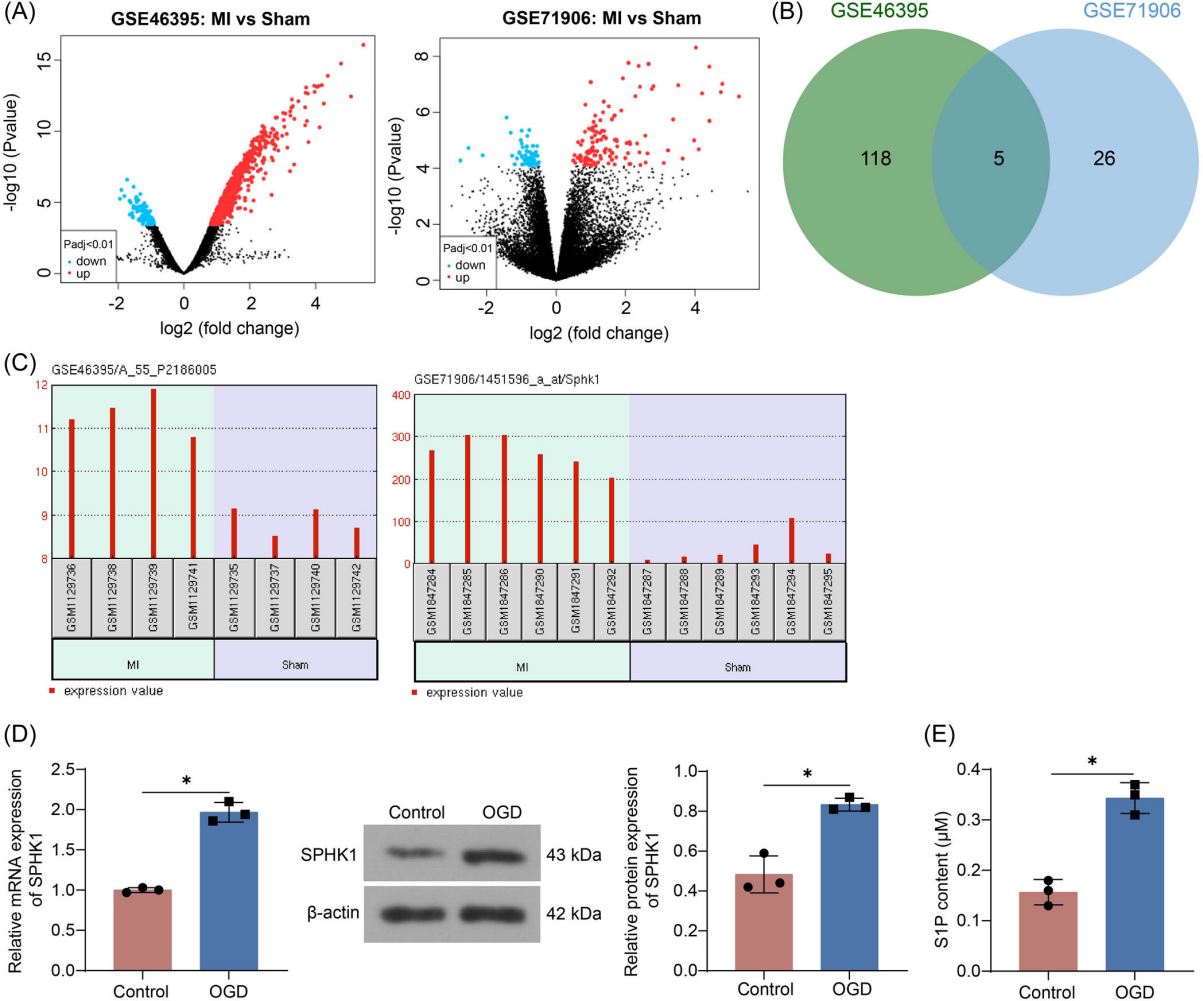
SPHK1 is upregulated in MI. (A) Volcano maps of differentially expressed genes in the GSE46395 and GSE71906 data sets. (B) The intersection of differentially expressed genes in the GSE46395 and GSE71906 data sets. (C) Expression of SPHK1 in MI in the GSE46395 and GSE71906 data sets. (D) SPHK1 mRNA and protein expression in OGD‐treated cells using RT‐qPCR and Western blot. (E) The content of S1P in OGD‐treated cells using ELISA. Results are expressed as the relative mean and SD ratio of three independent sets of experiments. Statistical significance was assessed by unpaired *t*‐test. *p < 0.05. mRNA, messenger RNA; MI, myocardial infarction; OGD, oxygen‐glucose deprivation; RT‐qPCR, reverse transcription‐quantitative polymerase chain reaction; S1P, Sphingosine‐1‐phosphate.

Differentially expressed genes in the GSE46395 and GSE71906 data sets were analyzed (Figure 1A). The genes differentially expressed in MI were screened with |Log2FC| > 2, adj *p* value < .01, and the two data sets were intersected. There were five intersected genes: HSPA1A, HBEGF, ANKRD2, SPHK1, and ADAMTS4 (Figure 1B). SPHK1 was highly expressed in MI in the GSE46395 and GSE71906 data sets (Figure 1C). The elevated mRNA and protein expression of SPHK1 was identified in HL‐1 cells exposed to OGD (Figure 1D). Further analysis using ELISA showed a significant increase in S1P content in HL‐1 cells treated with OGD (Figure 1E).

### 3.2 SPHK1 downregulation reduces cardiomyocyte injury

**Figure 2:**
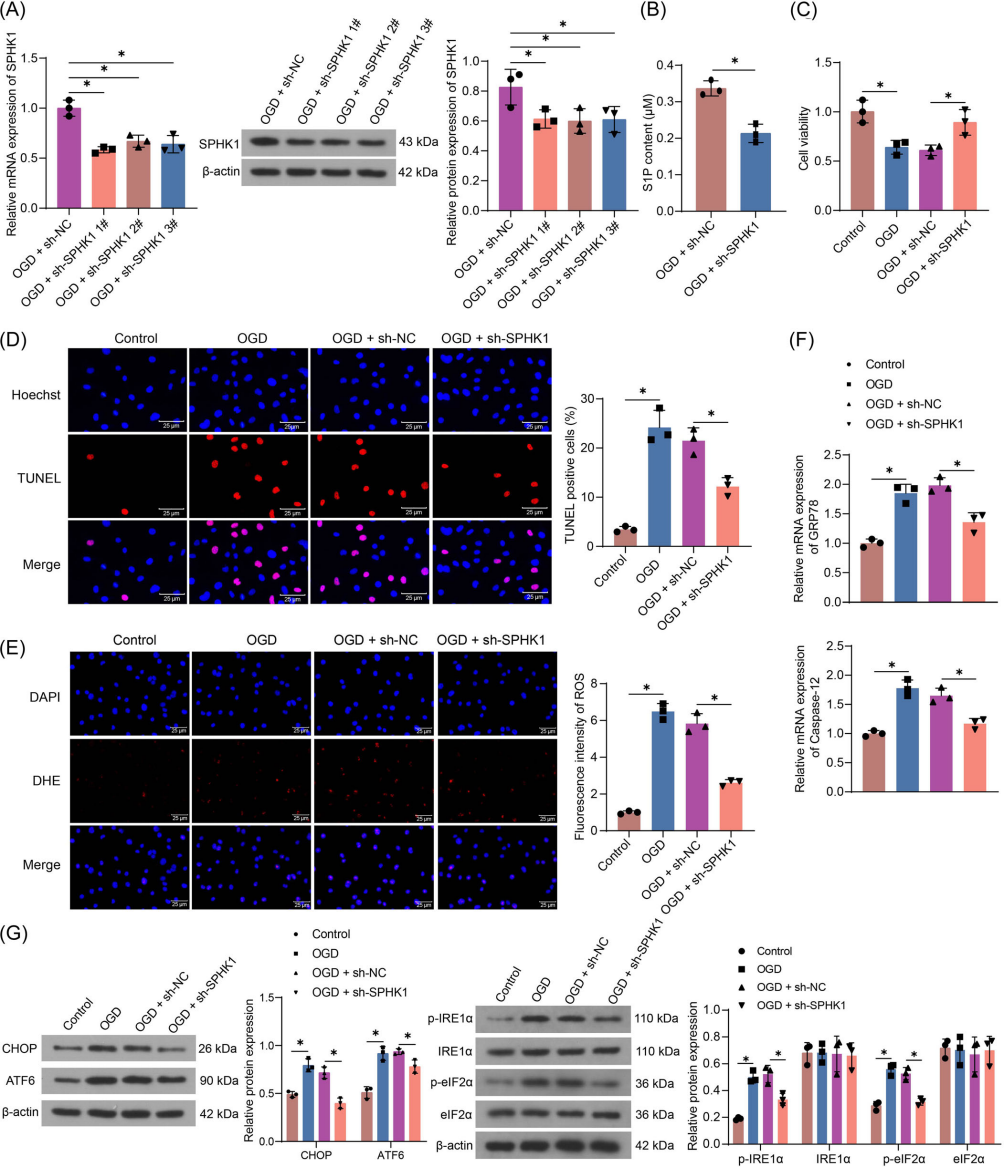
SPHK1 downregulation reduces ER stress and HL‐1 cell injury caused by OGD treatment. HL‐1 cells were transfected with sh‐SPHK1 or sh‐NC, followed by OGD. (A) Detection of SPHK1 expression in HL‐1 cells after SPHK1 downregulation by RT‐qPCR and Western blot. (B) The content of S1P in HL‐1 cells after SPHK1 downregulation using ELISA. (C) Cellular viability was examined using CCK‐8 assay. (D) Cell apoptosis was measured using TUNEL. (E) The ROS level of cells was evaluated using DHE staining. (F) The mRNA expression of GRP78 and Caspase‐12 in HL‐1 cells after SPHK1 downregulation was detected by RT‐qPCR. (G) The protein expression of CHOP and ATF6 as well as the extent of IRE1α and eIF2α phosphorylation in HL‐1 cells after SPHK1 downregulation was detected by western blot assays. Results are expressed as the relative mean and SD ratio of three independent sets of experiments. Statistical significance was assessed by one‐way ANOVA (A‐F) or two‐way ANOVA (G). *p < 0.05. ANOVA, analysis of variance; MI, myocardial infarction; mRNA, messsenger RNA; RT‐qPCR, reverse transcription‐quantitative polymerase chain reaction.

HL‐1 cells with SPHK1 knockdown were constructed by transfection of shRNAs and treated with OGD. Reduced expression of SPHK1 in HL‐1 cells was observed following the transfection of shRNAs (Figure 2A). The cells transfected with sh‐SPHK1#1 were selected for subsequent experimental analysis. ELISA confirmed that S1P content in cells decreased significantly with the downregulation of SPHK1 (Figure 2B). The cellular activity of HL‐1 cells, as determined using CCK‐8 assays, was reduced after OGD treatment. The viability of cells in the sh‐SPHK1 group was restored (Figure 2C). The TUNEL assay demonstrated that the apoptosis level of HL‐1 cells was augmented upon OGD, while the apoptosis rate of HL‐1 cells was decreased after SPHK1 downregulation (Figure 2D). ROS was increased after OGD treatment, while SPHK1 depletion reduced cellular oxidative stress to some extent (Figure 2E). The mRNA expression of GRP78 and Caspase‐12 was elevated after cells were challenged with OGD, which was reduced after SPHK1 loss (Figure 2F). Consistently, the protein expression of CHOP and ATF6 and the extent of IRE1α and eIF2α phosphorylation were upregulated in cells that were challenged with OGD and downregulated after SPHK1 knockdown (Figure 2G).

### 3.3 SPHK1 downregulation has a protective effect on MI mice

**Figure 3:**
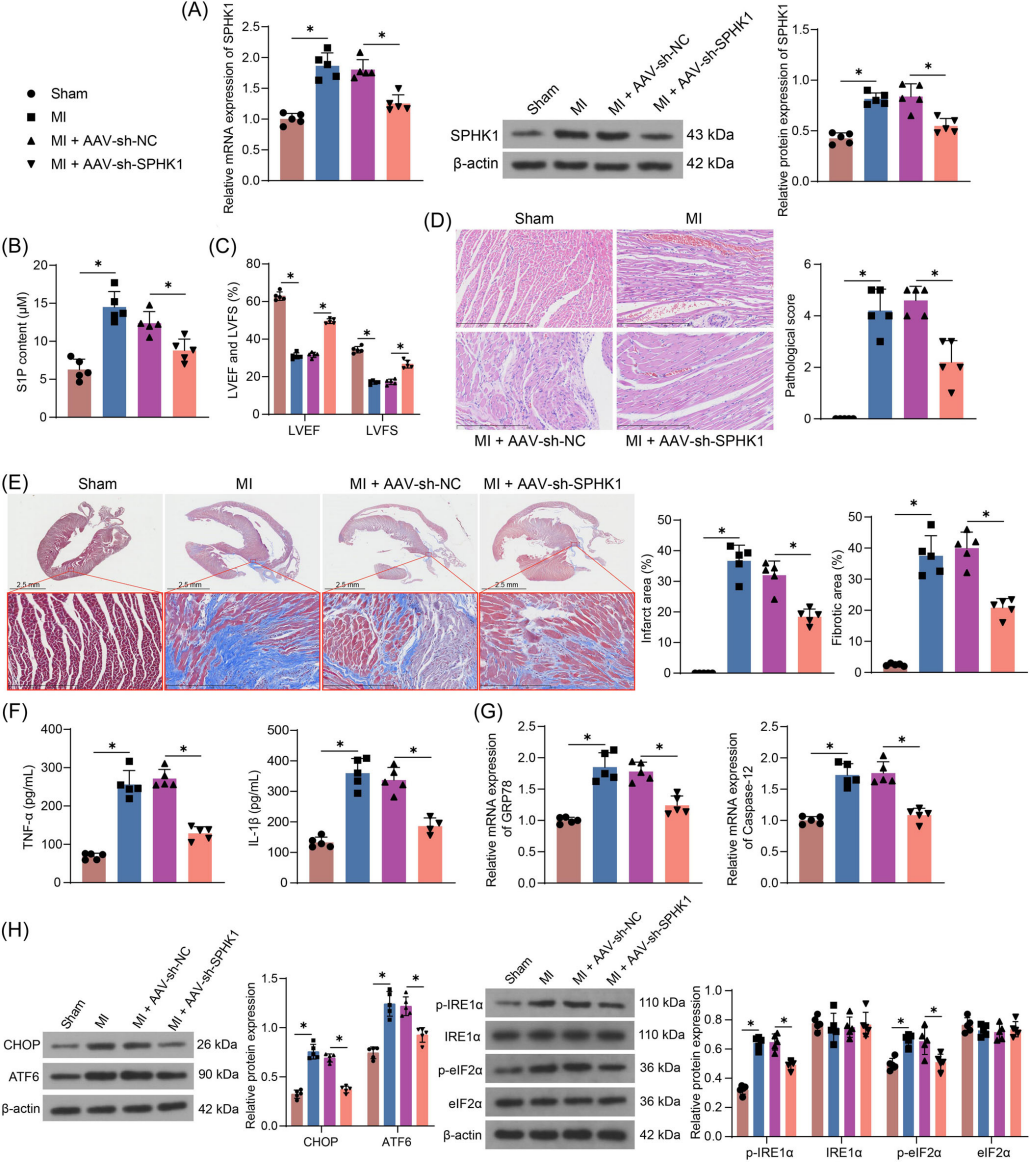
SPHK1 downregulation attenuates MI in mice. (A) Detection of SPHK1 expression in heart tissues of MI mice after SPHK1 downregulation using RT‐qPCR and Western blot. (B) The content of S1P in MI mice after SPHK1 downregulation using ELISA. (C) Echocardiographic examination of cardiac dysfunction in MI mice and MI mice after SPHK1 downregulation. (D) Pathological characteristics and scores of heart tissue in MI mice after SPHK1 downregulation detected by HE staining. (E) Detection of infarct size and myocardial fibrosis in heart tissues of MI mice after SPHK1 downregulation by Masson staining. (F) The determination of inflammatory factors (TNF‐α and IL‐1β) in mouse serum using ELISA. (G) The mRNA expression of GRP78 and Caspase‐12 in infarct margin tissues of mice was detected by RT‐qPCR. (H) The protein expression of CHOP and ATF6 as well as phosphorylation of IRE1α and eIF2α in infarct margin tissues of mice was detected by western blot assays. Results are expressed as the relative mean and SD ratio of the indicated number of animals (n = 5). Statistical significance was assessed by one‐way ANOVA (A, B, D‐G) and two‐way ANOVA (C, H). *p < 0.05. ANOVA, analysis of variance; MI, myocardial infarction; mRNA, messenger RNA; RT‐qPCR, reverse transcription‐quantitative polymerase chain reaction.

The MI mouse model was constructed by ligating LAD, and AAV‐sh‐NC or AAV‐sh‐SPHK1 were administrated into the ischemic hearts of MI mice, respectively. Elevated expression of SPHK1 in the hearts of MI mice and decreased expression of SPHK1 in the hearts of mice in the AAV‐sh‐SPHK1 group were observed (Figure 3A). Consistently, the contents of S1P were augmented in the hearts of MI mice, while downregulated by SPHK1 inhibition (Figure 3B). Echocardiography showed a significant reduction in LVEF and LVFS in mice. By contrast, the cardiac dysfunction of mice was alleviated upon the downregulation of SPHK1 (Figure 3C). It was revealed by HE staining that MI mice exhibited cardiomyocyte hypertrophy and inflammatory infiltration. Cardiomyocyte hypertrophy and inflammatory infiltration were alleviated in the mice treated with AAV‐sh‐SPHK1 compared with those administrated with AAV‐sh‐NC (Figure 3D). Masson staining demonstrated that the infarct area and myocardial fibrosis levels were increased in the MI mice relative to the sham‐operated mice. Compared with the AAV‐sh‐NC group, the infarct area and myocardial fibrosis levels were reduced in the AAV‐sh‐SPHK1 group (Figure 3E). The data from ELISA showed that the downregulation of SPHK1 reduced the levels of inflammatory factors (TNF‐α and IL‐1β) in the serum of MI mice (Figure 3F). Moreover, SPHK1 downregulation decreased the expression of GRP78 and Caspase‐12 at the mRNA level (Figure 3G). Downregulation of SPHK1 also alleviated the MI‐induced increase in protein expression of CHOP and ATF6 as well as phosphorylation levels of IRE1α and eIF2α (Figure 3H).

### 3.4 ATF3 regulates the transcriptional activity of SPHK1

**Figure 4:**
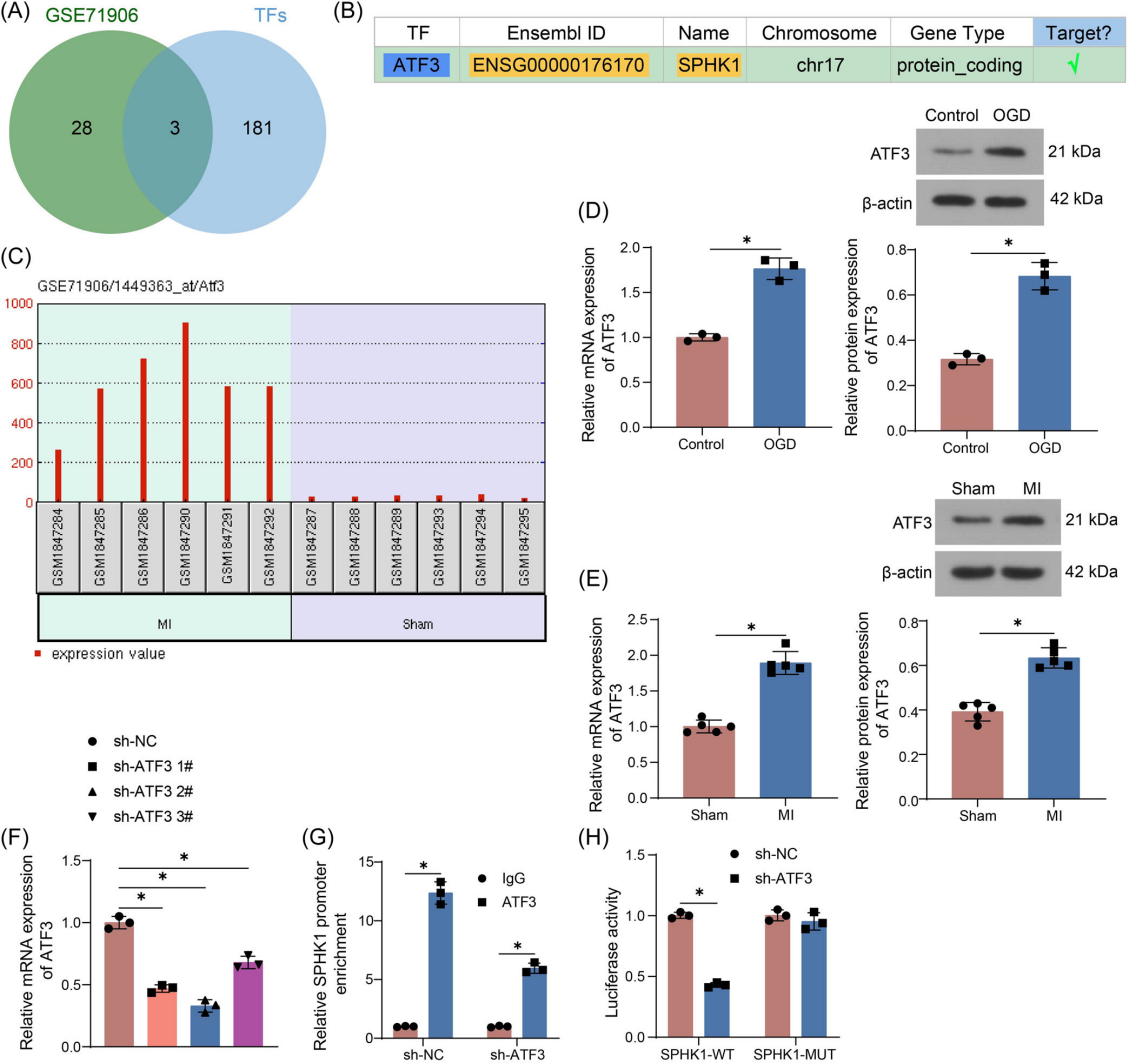
ATF3 targets SPHK1 in MI. (A) The intersection of transcriptional regulators of SPHK1 and differentially expressed genes in the GSE71906 data set. (B) The targeting relationship between ATF3 and SPHK1 predicted in hTFtarget. (C) Expression of ATF3 in MI in the GSE71906 data set. (D) Detection of ATF3 expression in OGD‐treated HL‐1 cells by RT‐qPCR and Western blot. (E) Detection of ATF3 expression in the heart of MI mice by RT‐qPCR and Western blot. (F) Detection of ATF3 expression in 293T cells after ATF3 downregulation by RT‐qPCR. (G) The enrichment of SPHK1 promoter on ATF3 using ChIP‐qPCR. (H) The transcriptional regulation of SPHK1 by ATF3 using dual‐luciferase assays. Results are expressed as the relative mean and SD ratio of the indicated number of animals (*n* = 5) or three independent sets of experiments. Statistical significance was assessed by unpaired *t*‐test (D, E), one‐way (F), or two‐way ANOVA (G and H). *p < 0.05. ANOVA, analysis of variance; MI, myocardial infarction; mRNA, messenger RNA; RT‐qPCR, reverse transcription‐quantitative polymerase chain reaction.

The transcriptional regulators of SPHK1 were queried in hTFtarget and intersected with the differentially expressed genes in the GSE71906 data set. Three intersected genes: ATF3, FOS, and MYC were found (Figure 4A). The most differentially expressed ATF3 in the GSE71906 data set was selected as the upstream target of SPHK1 (Figure 4B). ATF3 expression was shown to be elevated in MI in the GSE71906 data set (Figure 4C). Consistently, elevated expression of ATF3 was observed in OGD‐treated cardiomyocytes and heart tissues in MI mice (Figure 4D, E). sh‐ATF3 (#1, #2, and #3) was transfected into 293 T cells, and the expression of ATF3 was evaluated following the transfection (Figure 4F). sh‐ATF3 #2 was selected for subsequent experimental analysis based on RT‐qPCR results. Downregulation of ATF3 reduced the enrichment of the SPHK1 promoter sequence on ATF3 in 293 T cells, as revealed by ChIP‐qPCR (Figure 4G). The binding sites of ATF3 and SPHK1 promoters were predicted in hTFtarget for the construction of luciferase reporter gene vectors containing both WT and MUT binding sites. The dual‐luciferase results showed that ATF3 downregulation decreased the luciferase activity of SPHK1‐WT, whereas it did not impact the SPHK1‐MUT (Figure 4H).

### 3.5 SPHK1 inhibits the protective effect of ATF3 downregulation on cardiomyocytes

**Figure 5:**
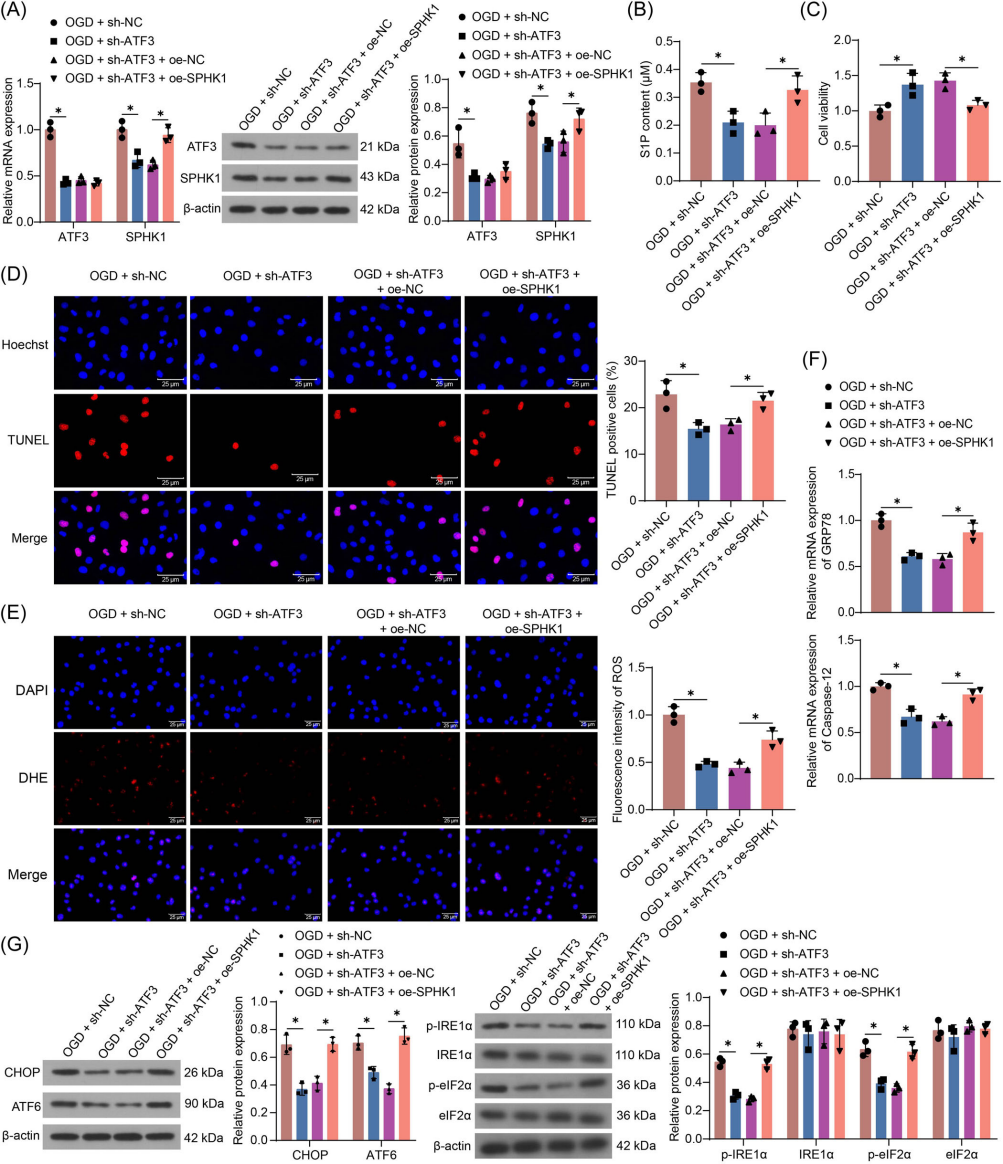
SPHK1 overexpression inhibits the protective effect of ATF3 downregulation on HL‐1 cell injury. HL‐1 cells were transfected with sh‐ATF3 (with sh‐NC as control) or cotransfected with sh‐ATF3 + oe‐SPHK1 (with sh‐ATF3 + oe‐NC as control) (A) Detection of ATF3 and SPHK1 expression in HL‐1 cells by RT‐qPCR and Western blot. (B) The content of S1P in HL‐1 cells using ELISA. (C) Cellular viability was examined using CCK‐8 assay. (D) Cell apoptosis was measured using TUNEL. (E) The ROS level of cells was evaluated using DHE staining. (F) The mRNA expression of GRP78 and Caspase‐12 in HL‐1 cells was detected by RT‐qPCR. (G) The protein expression of CHOP and ATF6 as well as phosphorylation of IRE1α and eIF2α in HL‐1 cells was detected by western blot assays. Results are expressed as the relative mean and SD ratio of three independent sets of experiments. Statistical significance was assessed by one‐way (B−F) or two‐way ANOVA (A, G). *p < 0.05. ANOVA, analysis of variance; MI, myocardial infarction; mRNA, messenger RNA; RT‐qPCR, reverse transcription‐quantitative polymerase chain reaction.

HL‐1 cells were transfected with sh‐ATF3 or cotransfected with sh‐ATF3 + oe‐SPHK1, followed by the OGD challenge. As expected, ATF3 and SPHK1 expression was decreased in HL‐1 cells that were transfected with sh‐ATF3 relative to those treated with sh‐NC. Relative to cells in the sh‐ATF3 + oe‐NC group, there was an insignificant difference in the expression of ATF3 in cells in the sh‐ATF3 + oe‐SPHK1 group, but the expression of SPHK1 was enhanced (Figure 5A). The content of S1P in the cells also decreased with the decrease of ATF3 expression and increased after overexpression of SPHK1 (Figure 5B). Moreover, the proproliferative and antiapoptotic effects of sh‐ATF3 on HL‐1 cells were compromised by SPHK1 overexpression (Figure 5C,D). Downregulation of ATF3 expression reduced oxidative stress in OGD‐treated HL‐1 cells, which was partially restored after SPHK1 overexpression (Figure 5E). The mRNA expression of GRP78 and Caspase‐12, the protein expression of CHOP and ATF6, as well as the extent of IREα and eIF2α phosphorylation were reduced in HL‐1 cells after ATF3 depletion, which was elevated after SPHK1 overexpression (Figure 5F,G).

### 3.6 SPHK1 aggravates myocardial injury in MI mice

**Figure 6:**
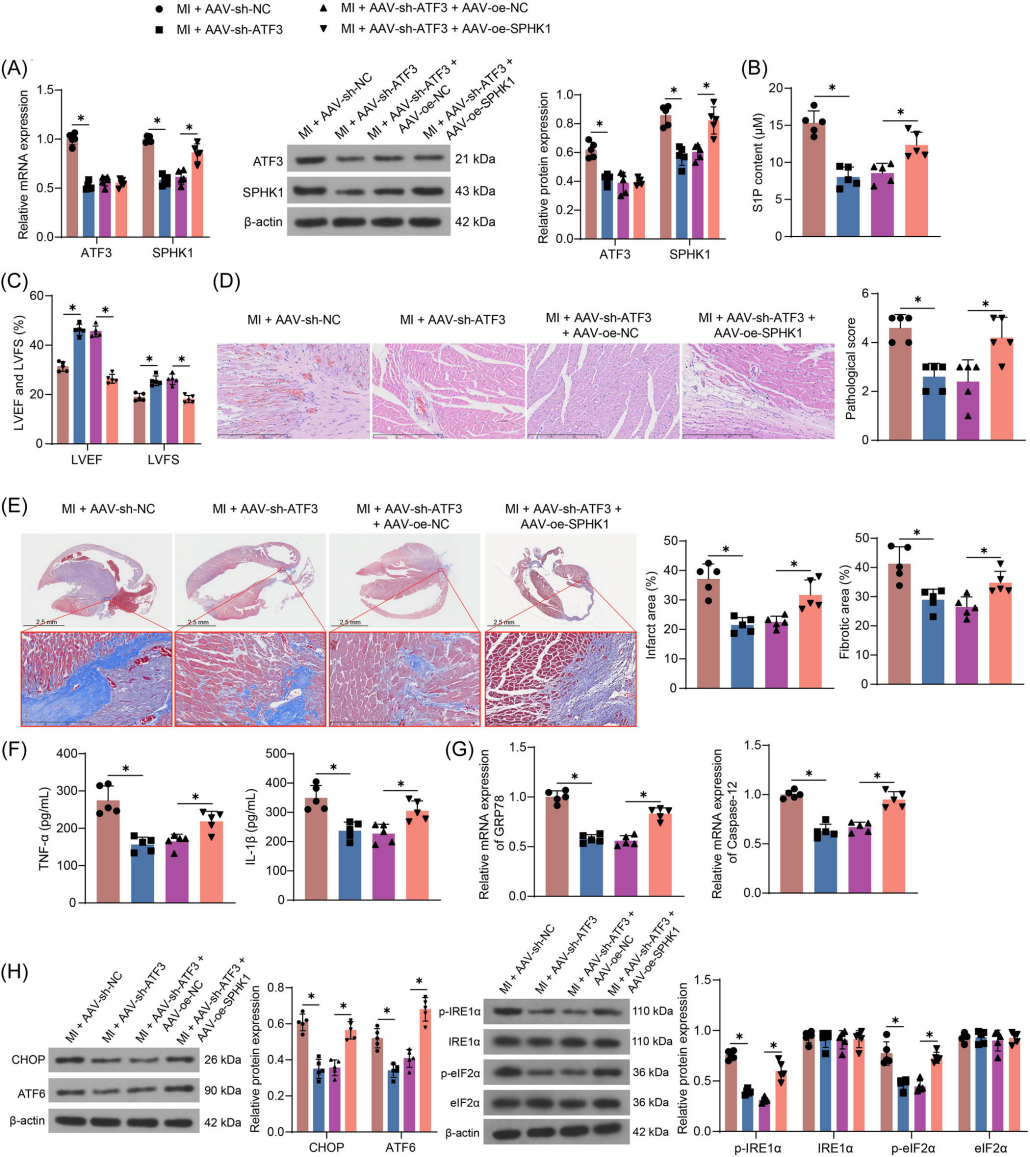
ATF3 downregulation attenuated MI in mice, and SPHK1 overexpression exacerbated MI. After MI treatment, the mice were injected with relevant AAV for gene expression manipulation. (A) Detection of SPHK1 and ATF3 expression in heart tissues of MI mice using RT‐qPCR and Western blot. (B) The content of S1P in MI mice using ELISA. (C) Echocardiographic examination of cardiac dysfunction in MI mice and MI mice. (D) Pathological characteristics and scores of heart tissue in MI mice detected by HE staining. (E) Detection of infarct size and myocardial fibrosis in heart tissues of MI mice by Masson staining. (F) The determination of inflammatory factors (TNF‐α and IL‐1β) in mouse serum using ELISA. (G) The mRNA expression of GRP78 and Caspase‐12 in infarct margin tissues of mice was detected by RT‐qPCR. (H) The protein expression of CHOP and ATF6 as well as phosphorylation of IRE1α and eIF2α in infarct margin tissues of mice was detected by western blot assays. Results are expressed as the relative mean and SD ratio of the indicated number of animals (n = 5). Statistical significance was assessed by one‐way (B, D‐G) or two‐way ANOVA (A, C, H). *p < 0.05. ANOVA, analysis of variance; MI, myocardial infarction; mRNA, messenger RNA; RT‐qPCR, reverse transcription‐quantitative polymerase chain reaction.

MI mice were further divided into 4 groups by AAV injection: the MI + AAV‐sh‐NC, MI + AAV‐sh‐ATF3, MI + AAV‐sh‐ATF3 + AAV‐oe‐NC and MI + AAV‐sh‐ATF3 + AAV‐oe‐SPHK1 groups (n = 5). The expression of ATF3 and SPHK1 in the heart tissues of mice was measured. The expression of ATF3 and SPHK1 in the heart tissues of mice in the MI + AAV‐sh‐ATF3 group was reduced relative to the MI mice administrated with AAV‐sh‐NC. Relative to the MI + AAV‐sh‐ATF3 + AAV‐oe‐NC group, insignificant differences in the expression of ATF3 and elevated expression of SPHK1 were noted in the heart tissues of mice in the MI + AAV‐sh‐ATF3 + AAV‐oe‐SPHK1 group (Figure 6A). Meanwhile, S1P content in mouse hearts also decreased with the decrease of ATF3, and S1P content was significantly restored after overexpression of SPHK1 (Figure 6B). Cardiac dysfunction in MI mice was also alleviated with a decrease in ATF3, whereas upregulation of SPHK1 reversed this phenomenon, with a significant decrease in LVEF and LVFS values in mice (Figure 6C). HE staining showed that loss of ATF3 expression alleviated cardiomyocyte hypertrophy and inflammatory infiltration in mice, which was accentuated after SPHK1 overexpression (Figure 6D). Consistently, infarct size and myocardial fibrosis levels were reduced in mice with ATF3 downregulation, while SPHK1 overexpression decreased the anti‐fibrotic effect of ATF3 downregulation in MI mice (Figure 6E). In addition, ATF3 downregulation decreased inflammation in MI mice, and SPHK1 overexpression played a proinflammatory role in mice with MI (Figure 6F). The expression of ER stress‐related genes was reduced in MI mice after ATF3 downregulation and SPHK1 overexpression suppressed the repressive effect of ATF3 downregulation on ER stress, as evidenced by the restored mRNA expression of GRP78 and Caspase‐12, protein expression of CHOP and ATF6, as well as the IRE1α and eIF2α phosphorylation (Figure 6G,H).

## 4 DISCUSSION

Oxidative stress, cytokine overproduction, massive cardiomyocyte death, as well as ATP metabolism in cardiomyocytes have been identified as possible molecular mechanisms underlying MI.^19^, ^20^, ^21^ Interestingly, ischemia/reperfusion injury has been linked to ER stress, whereas prolonged ER stress contributed to cardiac hypertrophy and heart failure.^22^ Here, we observed that both LAD ligation and OGD treatment upregulated SPHK1 expression in cardiomyocytes, along with increasing ER stress. Disruption of SPHK1 markedly improved myocardial fibrosis and cardiac dysfunction after MI and attenuated ER stress and inflammatory response in cardiomyocytes. Mechanistically, ATF3 targets and positively regulates the transcription of SPHK1. SPHK1 overexpression blocked sh‐ATF3‐alleviated ER stress and inflammation Therefore, ATF3 activation facilitates ER stress‐induced apoptosis through SPHK1.

Even though Karliner et al. showed the protecting effects of S1P and SPHK2 on mouse myocardium against ischemia/reoxygenation injury,^23^, ^24^ the present work mainly focused on its role in ER stress since SPHK1 deficiency has been revealed to prevent mice against ER stress and mitochondrial permeability transition.^25^ ER stress, caused by the dysregulation of ER Ca^2+^ levels, induces the production of Ca^2+^‐binding ER chaperones, including GRP78.^26^ In this study, we also observed the downregulation of GRP78, Caspase‐12, CHOP, and ATF6 expression, and IRE1α and eIF2α phosphorylation in response to the silencing of SPHK1. This ample evidence preliminarily validated the regulatory role of SPHK1 in ER stress. SPHK1 has been recently summarized to promote survival and cell proliferation and hamper inflammation.^27^ Under the condition of hepatic ischemia/reperfusion injury, SPHK1 knockout mice were found to have alleviated inflammation and oxidative stress relative to WT mice, as manifested by lower IL‐1β, TNF‐α, MPO, and MDA contents.^28^ These reports were consistent with our observation that SPHK1 depletion lowered ROS production in the HL‐1 cells and the contents of IL‐1β and TNF‐α in the serum of MI mice. Mice with MI exhibit replacement fibrosis resulting from tissue injury, myocyte death, and inflammation.^29^ Pchejetski et al. reported that Apelin, an adipocyte‐derived factor, can protect cardiac fibroblasts from activation and collagen overproduction by inhibiting SPHK1.^30^ The suppression of SPHK1 using PF543, an inhibitor of SPHK1, decreased the mitochondrial ROS in lung fibroblasts and the expression of alpha‐SMA.^31^ The knockdown of SPHK1 increased survival and resistance to pulmonary fibrosis in bleomycin‐challenged mice.^32^ In addition, TGF‐β1 evoked the activation of SPHK1, increased intracellular S1P, and upregulated expression of SPHK1, Col a1(I), and Col a1(III).^33^ Therefore, the pro‐fibrotic properties of SPHK1 were substantiated, and the specific downstream pathway through which SPHK1 regulated myocardial fibrosis awaits further exploration.

By combining the data from the GSE71906 data set and the predicted list of upstream transcription factors of SPHK1 in the hTFtarget website, we obtained three possible candidates: ATF3, FOS, and MYC. Intriguingly, in a transcriptional regulatory network of differentially expressed genes in the MI‐related GSE4648 data set, these three transcription factors were also found.^34^ Among them, ATF3, showing the largest differential expression in the GSE71906 data set was chosen for further analysis in this study. It has been established by Kalfon et al. that mice with JDP2 and ATF3 depletion had alleviated maladaptive cardiac remodeling and lower hypertrophy upon transverse aortic constriction, indicating that inhibition of ATF3 and JDP2 is beneficial for cardiac function.^35^ Koren et al. also showed that ATF3 overexpression in mice contributed to ventricles hypertrophy, heart dysfunction, as well as fibrosis,^36^ and later reported that CXCR3 was positively mediated by ATF3 via an ATF3 transcription response element in its proximal promoter during cardiac remodeling.^37^ ATF3, a stress‐induced transcription factor, controls metabolism, immunity, as well as oncogenesis,^38^ and various extracellular signals, such as ER stress and cytokines have been related to ATF3 overproduction.^39^ Furthermore, silencing of ATF3 downregulated the expression of pro‐fibrotic genes and impaired the hepatic stellate cell activation, thereby improving liver fibrosis, representing a protective role of ATF3 inhibition against fibrosis.^40^ Here, we observed that loss of ATF3 using shRNAs showed identical suppressing effects on ER stress and inflammatory response as sh‐SPHK1, which was overturned by SPHK1 overexpression.

This study had several limitations. First, the activation of SPHK1 and the ensuing S1P production in the serum of mammals represent a main checkpoint in many cellular signaling,^41^ and the SPHK1/S1P/S1P receptor 1 (S1PR1) axis induction attributed to inflammatory responses in cardiomyocytes.^42^ In addition, Ahmed et al. suggested that S1PR1 expressed strongly and were uniformly distributed in all chambers of the heart with no remarkable difference in human and rat myocardial tissues, while S1PR2, S1PR3, S1PR4, and S1PR5 expression was either weak or not detectable in both human and rat heart.^43^ Taken together, these findings could suggest that S1P‐mediated downstream signaling in cardiac tissues may be primarily associated with S1PR1. Second, we only perform molecular investigations on SPHK1, and the functional role of SPHK2 should be studied in our following studies. Third, ATF3, FOS, and MYC were predicted to be transcriptional regulators of SPHK1 in the present study. Even though FOS with siRNAs has been reported to substantially inhibit the expression of SPHK1 in glomerular mesangial cells exposed to high glucose ^44^ and SPHK1 overexpression stimulated intestinal epithelial cell proliferation through increasing MYC translation,^45^ whether there are similar regulatory mechanisms in MI remains to be further elucidated.

## 5 CONCLUSION

In summary, stimulation of ATF3 exerted pro‐fibrotic and proinflammatory effects and expedited ER stress in cardiomyocytes via SPHK1. Thus, the inhibition of ATF3 and SPHK1 may have therapeutic potential for MI.

## AUTHOR CONTRIBUTIONS

**Huiling Chen**: Conceptualization; data curation; validation; writing—original draft. **Suxin Luo**: Data curation; formal analysis, validation. **Cong Zhang**: Validation, methodology, resources. **Huamei Chen**: Validation, data curation, writing—review and editing.

## CONFLICT OF INTEREST STATEMENT

The authors declare no conflict of interest.

## Data Availability

Data are available on request due to privacy/ethical restrictions.
